# Aphtha

**Published:** 1849-09

**Authors:** J. Yale Ware

**Affiliations:** Mass.


*Aphtha.* By J. Yale Ware, Mass.—The following simple prescription has proved a specific in my hands in many hundred cases of aphtha. I learned it of Dr. Eli Ives, New Haven. ℞.—Ipecac. gr. vi; tinct. opii; ess. pep., aa gtt. iv; boiling water xxiv teaspoonfuls. Sweeten with loaf sugar. Dose, a teaspoonful every two hours. At the same time apply to the tongue equal parts of a powder of borax and loaf sugar, which the child will carry over the mouth. I have never known the above to fail in any case of infants' sore mouth. It generally cures in two days. Occasionally, in delicate subjects, the disease returns again, when the remedy needs repeating.

